# The Mediating Role of Psychological Inflexibility in the Relationship Between Social Appearance Anxiety and Problematic Social Media Use Among Adolescents

**DOI:** 10.3390/children13081091

**Published:** 2026-08-17

**Authors:** Canan Bal, Eyüp Çelik, Elife Nur Saydam

**Affiliations:** 1 Department of Guidance and Psychological Counseling, Faculty of Education, Sakarya University, Sakarya 54300, Türkiye; canan.bal@ogr.sakarya.edu.tr (C.B.); eyupcelik@sakarya.edu.tr (E.Ç.); 2 Department of Primary Education, Faculty of Education, Sakarya University, Sakarya 54300, Türkiye

**Keywords:** adolescent, psychological inflexibility, social appearance anxiety, problematic social media

## Abstract

**Highlights:**

**What are the main findings?**

- Social appearance anxiety was positively associated with problematic social media use and psychological inflexibility among adolescents.
- Psychological inflexibility partially mediated the association between social appearance anxiety and problematic social media use.
- Social appearance anxiety was significantly associated with problematic social media use both directly and indirectly through psychological inflexibility.

**What are the implications of the main finding?**

- The partial mediating role of psychological inflexibility in the association between social appearance anxiety and problematic social media use highlights psychological inflexibility as a potential intervention target.
- Prevention and intervention programs targeting problematic social media use among adolescents should consider social appearance anxiety and psychological inflexibility.

**Abstract:**

**Background/Objectives**: Problematic social media use (PSMU) has become an increasingly important mental health concern among adolescents and young adults. This study aimed to examine the mediating role of psychological inflexibility (PI) in the relationship between social appearance anxiety (SAA) and problematic social media use (PSMU) among adolescents. **Methods**: Data were collected from 491 high school students (334 girls, 68%; 157 boys, 32%) in the city of Sakarya, Turkey, who were enrolled in different grade levels and selected on a voluntary basis. The Social Appearance Anxiety Scale (SAAS), the Avoidance and Fusion Questionnaire for Youth–8 (AFQ-Y8), and the Social Media Addiction Scale for Adolescents (SMSA) were used to collect the data. The relationships among PSMU, SAA, and PI were analyzed using structural equation modeling with AMOS 24. **Results**: Significant positive relationships were found among PSMU, SAA, and PI. Mediation analysis indicated that psychological inflexibility partially mediated the relationship between SAA and PSMU. Moreover, psychological inflexibility was significantly associated with problematic social media use. SAA showed both direct and indirect associations with problematic social media use through psychological inflexibility. **Conclusions**: These findings suggest that psychological inflexibility may partially explain the association between SAA and PSMU and highlight its potential as a target for future prevention and intervention efforts.

## 1. Introduction

Owing to its diverse applications across several fields, social media has experienced rapid growth in usage and has become a necessary part of everyday life. According to Statista [1], approximately 5.56 billion people use social media platforms, representing 67.9% of the global population. However, social media use can also exert adverse physical, psychological, and behavioral effects, a phenomenon termed PSMU [2]. As social media adoption continues to expand worldwide, there has been a remarkable rise in the utilization of network applications among adolescents [3,4]. Adolescence, characterized by distinct neurobiological, social, and cognitive development and spanning the period between childhood and adulthood, is one of the most widely researched stages of human development [5]. As adolescents engage heavily with social media, research focused on protecting young people from the risks linked with problematic use is essential.

Given the widespread use of social media during adolescence, understanding the factors associated with problematic social media use has become an important research priority. Based on Jiang et al. [6], problematic social media use has not yet been part of the clinical categories of mental disorders in the DSM. Furthermore, researchers differ regarding terminology, and there is no clear consensus on specific descriptors; consequently, the term “PSMU” is often preferred over “addiction” [3]. Due to existing conceptual confusion regarding the classification of PSMU, there is no consensus among researchers on the definition of problematic social media use [7,8]. PSMU is not defined solely by the amount of time spent on social media; it also includes symptoms such as excessive focus on social media, mood changes, an increased urge to use, withdrawal-like distress, unsuccessful attempts to reduce usage, and conflict with other areas of life [5]. In their study, Faverio and Sidoti [9] note that visually oriented social media platforms such as YouTube, TikTok, Instagram, and Snapchat are quite widespread among adolescents; previous research also shows that adolescents frequently use more than one social media platform. Data also indicates that the Facebook platform is widely used among adolescents, particularly in the United States [10]. Social media is frequently used by adolescents—particularly during adolescence—to communicate with peers, maintain social relationships, express themselves, and develop their identity. It can be said that social media offers adolescents digital spaces where they can gain new experiences related to their interests, join different communities, and find social support, and that adolescents frequently use it for these purposes.

A study conducted by Boer et al. [11] across 29 countries exhibited that 7% of adolescents aged 11–15 have been observed with high levels of PSMU. Furthermore, PSMU has been connected with lower subjective well-being [12] and depressive symptoms [13]. Moreover, Lopes et al. [14] have also revealed relationships with both depression and anxiety in their research. PSMU is often employed as a strategy to avoid anxiety-inducing emotions, thoughts, and experiences as well [15]. While excessive SMU in some countries has been linked to increased social participation, peer support, and life satisfaction, problematic use consistently reduced adolescents’ quality of life in every aspect [11]. A young person’s problematic use of social media can also affect their perceptions of their own physical appearance.

SAA is a prevalent condition frequently observed among adolescents. It is conceptualized as fear experienced by individuals and anxiety regarding the potential negative evaluation of their physical appearance by their social environment [16]. Individuals with high levels of SAA tend to avoid close interpersonal relationships and maintain greater distance from others [17]. In addition, social appearance anxiety has been reported to have negative effects on individuals’ social, academic, and professional functioning [18]. Factors associated with SAA levels among adolescents are social media content and user interactions [19,20], PSMU [3,21,22,23] and being exposed to short-form videos on social media [24]. Some studies have yielded conflicting results, indicating that social media can function as both beneficial and harmful influences on SAA [25]. Although most studies have examined PSMU as a predictor of SAA, emerging evidence suggests that SAA may also contribute to PSMU.

The rise in appearance-related concerns among adolescents, alongside the widespread use of social media, has made it necessary to understand the link between SAA and PSMU. Research attributes the rise in SAA to the intensity of social media usage [26]. Some studies have found that excessive social media use is associated with body image dissatisfaction [27]. Furthermore, findings state that individuals who receive positive feedback on content shared on social media may experience a temporary improvement in their perceived social appearance, which reinforces addictive behaviors [28]. Although significant relationships have been established, these do not necessarily imply direct causation. Most previous studies have examined PSMU as a predictor of SAA. However, Boursier et al. [3] reported that SAA predicted PSMU among adolescent boys. Therefore, SAA may also be considered a potential predictor of PSMU.

Psychological inflexibility, one of the key concepts of Acceptance and Commitment Therapy (ACT), refers to an individual’s inability to engage in behaviors consistent with their long-term values or to modify their behavior when situational conditions require change [29]. PI encompasses six interrelated processes: cognitive fusion, experiential avoidance, present-moment disconnection, self-as-content, lack of contact with values, and inaction (or uncommitted action) [29,30]. It is generally conceptualized as the opposite of psychological flexibility, reflecting a rigid pattern of responding to internal experiences that limits adaptive functioning [29,30]. Chou and colleagues [31] demonstrated that PI is positively associated with both the severity of mental health problems and internet addiction. Consistent with this, individuals with lower levels of psychological flexibility were more likely to exhibit problematic internet use [32].

The literature has shown that reduced psychological flexibility, including experiential avoidance and cognitive fusion, is associated with appearance-related concerns [33]. As these processes constitute core components of PI, this finding provides indirect support for the association between PI and appearance-related concerns. Individuals with higher levels of PI may experience greater difficulties in accepting negative internal experiences and may engage in greater cognitive fusion with distressing self-evaluations [30]. Indeed, studies have demonstrated that higher levels of PI are associated with increased levels of SAA [34]. Moreover, PI has been shown to exacerbate the impact of appearance-related ideals on body image disturbances and disordered eating, highlighting its role in appearance-related psychological difficulties [35]. Accordingly, PI may represent an important psychological factor contributing to the maintenance of SAA by leading individuals to develop rigid responses toward appearance-related negative thoughts and emotions.

Studies have shown that the core processes of psychological inflexibility are associated with maladaptive coping strategies in individuals with appearance-related concerns [36]. It can be argued that individuals who have had negative experiences regarding their appearance may experience higher levels of psychological inflexibility, as difficulties in accepting and regulating appearance-related thoughts and emotions may limit their ability to respond flexibly to these experiences. Individuals with problematic internet use also tend to report feeling more anxious about their social appearance [37]. Based on these findings, the hypothesis has been put forward that PI is associated with PSMU and SAA and may play a mediating role between these two variables. Examining the role of PI in the relationship between SAA and PSMU may provide information about the processes underlying the effect of SAA on PSMU.

### 1.1. Theoretical Framework

During adolescence, concerns about appearance become a major source of anxiety for teenagers [38]. At the same time, adolescents increasingly seek social acceptance based on their physical appearance, and together with the significant physical changes that occur during this developmental period, this may increase their sensitivity to others’ evaluations, thereby contributing to the development of social appearance anxiety [38,39]. According to Acceptance and Commitment Therapy (ACT), individuals tend to avoid distressing thoughts and emotions while simultaneously becoming cognitively fused with these experiences, perceiving them as absolute truths [29]. Experiential avoidance and cognitive fusion are core processes of PI and may hinder individuals’ ability to respond flexibly to negative internal experiences [29]. Accordingly, adolescents experiencing high levels of SAA may become cognitively entangled with negative evaluations of their appearance and may try to avoid the distress caused by these evaluations. This avoidant coping style in response to anxiety can lead to an increase in the individual’s level of PI [40]. Indeed, stress has been proposed to motivate individuals toward digital escapism, thereby contributing to PSMU [41]. Adolescents with high levels of SAA may therefore use social media to avoid concerns about negative evaluations of their appearance or to obtain temporary emotional relief. From this perspective, ACT provides a comprehensive theoretical framework for explaining the potential relationships among SAA, PI, and PSMU.

Although previous studies have found associations among SAA, PI, and PSMU, to our knowledge, no study has examined whether PI mediates the relationship between SAA and PSMU among adolescents. Investigating this relationship may enhance understanding of PSMU and contribute to more effective interventions.

### 1.2. Hypotheses

**H1.**  *There is a significant positive relationship between SAA, PI, and PSMU.*

**H2.**  *PI mediates the positive relationship between SAA and PSMU.*

**Figure 1:**
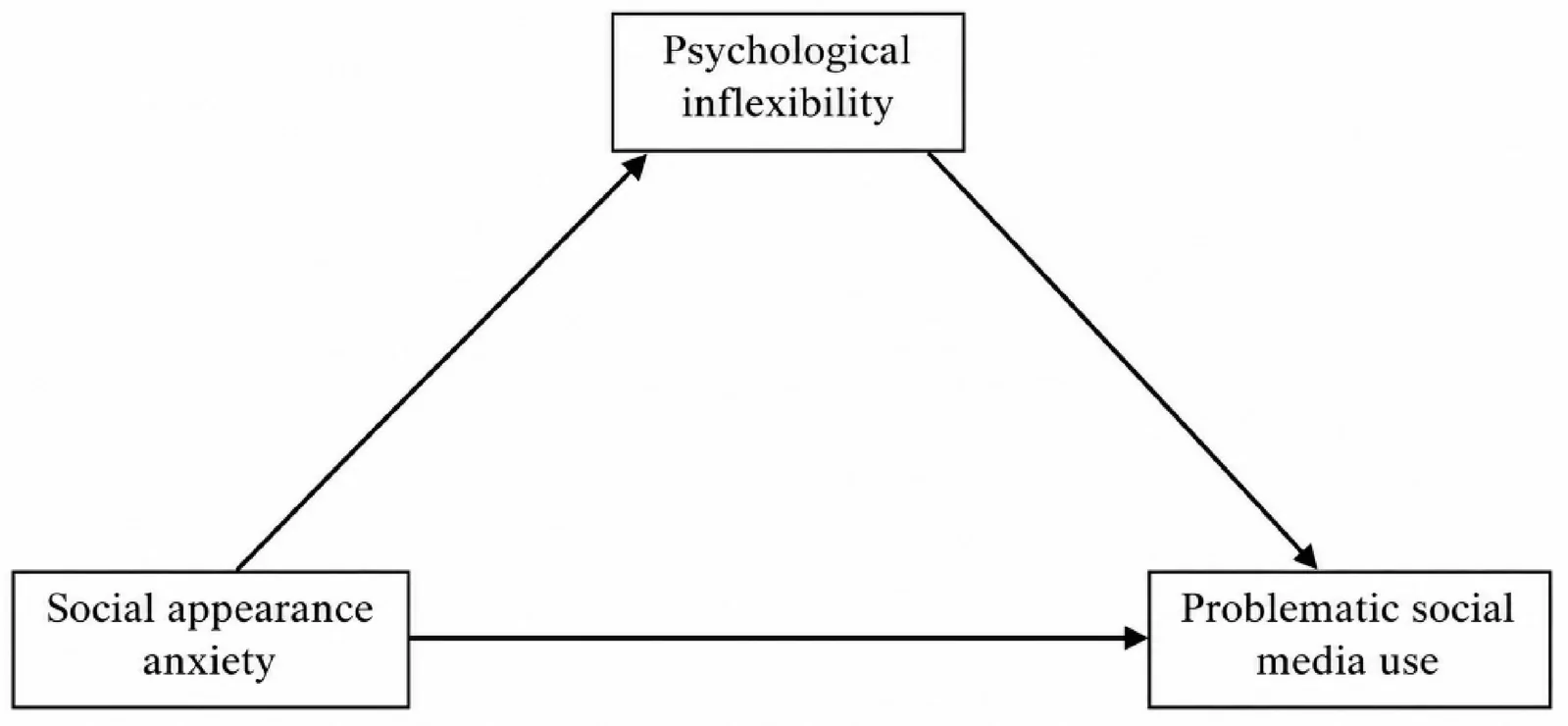
The Hypothesized Model.

The hypothesized model is presented in Figure 1.

## 2. Methods

In this study, which examines the mediating role of PI in the relationship between PSMU and SAA, a correlational research design has been adopted.

### 2.1. Participants and Procedure

The study participants, selected through convenience sampling, consisted of a total of 491 high school students; 334 of them (68%) were female, and 157 (32%) were male. The high school students participating in the study attend various high schools in the Hendek district of Sakarya Province, Turkey. The participants were enrolled in grades 9 to 12 and corresponded to an expected age range of 14 to 18 years. Regarding grade-level distribution, 140 students (28.5%) were in the 9th grade, 127 students (25.9%) were in the 10th grade, 94 students (19.1%) were in the 11th grade, and 130 students (26.5%) were in the 12th grade. Students were informed that participation in the study was voluntary; participants were selected accordingly on a voluntary basis. For minors, parental consent was obtained from their families regarding their participation in the study. Participants who were 18 years old provided informed consent on their own behalf. The measurement scales used for data collection were digitized and administered to the participating students via Google Forms. The data collection process was completed between 8 May 2025 and 8 June 2025. None of the surveys administered to the students were deemed invalid; all of the data collected were included in the analysis. To ensure confidentiality and anonymity, numerical identification numbers were assigned to participants. The principles of confidentiality and scientific integrity were strictly adhered to throughout the research.

Ethical approval was obtained from the Ethics Committee of Sakarya University, the institution to which the authors are affiliated, to conduct this study (Approval Date: 9 April 2025; Approval No.: E-61923333-050.99-46579).

### 2.2. Measurement Tools

#### 2.2.1. Social Appearance Anxiety Scale (SAAS)

The SAAS is a 16-item, 5-point scale and is unidimensional; it was developed by Hart et al. [16] to measure individuals’ social anxiety regarding their appearance. The scale was adapted into Turkish by Doğan [42], and Exploratory Factor Analysis (EFA) was confirmed by Confirmatory Factor Analysis (CFA). The EFA results indicated that the eigenvalue was 8.49 and that the single-factor structure explained 53.4% of the total variance. Factor loadings ranged from 0.35 to 0.87. In the CFA, the model fit indices were found to be significant (χ^2^ = 143.79, *N* = 254, *p* = 0.01). The Turkish version of the scale demonstrated excellent internal consistency (Cronbach’s α = 0.93). In the present study, Cronbach’s alpha coefficient was 0.95. The response options range from “Completely appropriate” to “Not at all appropriate”. The first item of the scale is reverse-scored, with high scores indicating high SAA. The test–retest reliability coefficient of the scale is 0.85.

#### 2.2.2. Avoidance and Fusion Questionnaire for Youth–8 (AFQ-Y8)

Developed by Greco et al. [43], this tool measures experiential avoidance and cognitive fusion in youth. The Turkish short form was adapted by Büyüköksüz and Erözkan [44]. The 8-item scale uses a 5-point Likert-type format (4: Very true 0: Not at all true). Total scores range from 0 to 32, with higher scores signifying increased avoidance and fusion. CFA results for the Turkish adaptation (KBÖ-G8) showed a well-fitted unidimensional structure (χ^2^/df = 2.18, CFI = 0.97, GFI = 0.97, NFI = 0.95, RMSEA = 0.06, SRMR = 0.04). Data from 325 high school students revealed an internal consistency (α = 0.78) and a two-week test–retest reliability of 0.79. In the present study, Cronbach’s alpha coefficient was 0.86. In the present study, psychological inflexibility was operationalized using the AFQ-Y8 total score, which reflects two central processes of psychological inflexibility: experiential avoidance and cognitive fusion. Although psychological inflexibility is conceptualized within ACT as a broader construct involving six interrelated processes [29], the AFQ-Y8 primarily assesses these two core components. Therefore, the present study focuses on these two processes rather than claiming to comprehensively assess all ACT processes.

#### 2.2.3. Social Media Addiction Scale for Adolescents (SMASA)

Constructed by Özgenel et al. [45], the SMASA evaluates social media addiction based on DSM-5 gaming addiction criteria. This 9-item unidimensional scale utilizes a 5-point Likert format, ranging from 5 (Always) to 1 (Never). A sample item is “I use social media more to feel happy.” No items are reverse-coded. Scores range from 9 to 45; higher scores show higher addiction. EFA yielded a single-factor structure explaining 56.787% of the variance. Criterion-related validity was supported by a significant positive correlation with the Game Addiction Scale (*r* = 0.554). Cronbach’s alpha was 0.90 using a sample of 634 students. In the present study, Cronbach’s alpha coefficient was 0.85. Although the Social Media Addiction Scale for Adolescents was originally developed to assess social media addiction, it has been used in previous studies to assess PSMU [46]. Therefore, in the present study, scale scores were interpreted as indicators of problematic social media use rather than clinical addiction.

### 2.3. Statistical Analysis

Interrelationships among variables were analyzed using structural equation modeling (SEM) in AMOS 24. The SEM analyses were conducted using the maximum likelihood (ML) estimation method. No missing data were identified in the dataset. χ^2^/df, CFI, TLI, IFI, GFI, AGFI, and RMSEA were used to evaluate model fit. Before testing the structural model, a three-factor measurement model was evaluated using confirmatory factor analysis (CFA). The CFA model was assessed based on χ^2^, degrees of freedom (df), *p*-value, RMSEA with its 90% confidence interval, CFI, TLI. Standardized factor loadings and correlations among latent factors were also examined to evaluate the adequacy of the measurement model. To improve the measurement model fit, error covariances between conceptually related items of the Social Appearance Anxiety Scale were specified based on the modification indices provided by AMOS. Specifically, post hoc error covariances were added between SAAS items 2 and 3 and between SAAS items 11 and 14. These modifications were made only when they were theoretically justifiable, as these item pairs assess closely related aspects of social appearance anxiety, consistent with recommendations in the structural equation modeling literature [47,48]. No other modification indices were incorporated into the final model. The mediation analysis was tested using bootstrapping with (5000) resamples, and bias-corrected 95% confidence intervals (CIs) were calculated to evaluate the significance of indirect effects. Statistical significance was set at *p* < 0.05. A Monte Carlo power analysis was conducted using the Monte Carlo Power Analysis application [49]. Based on the observed path coefficients and the study sample size (*N* = 491), the statistical power was estimated to be greater than 0.99.

## 3. Results

Before conducting the mediation analysis, a correlation analysis was performed to examine the relationships among the study variables (Table 1).

### 3.1. Measurement Model

**Figure 2:**
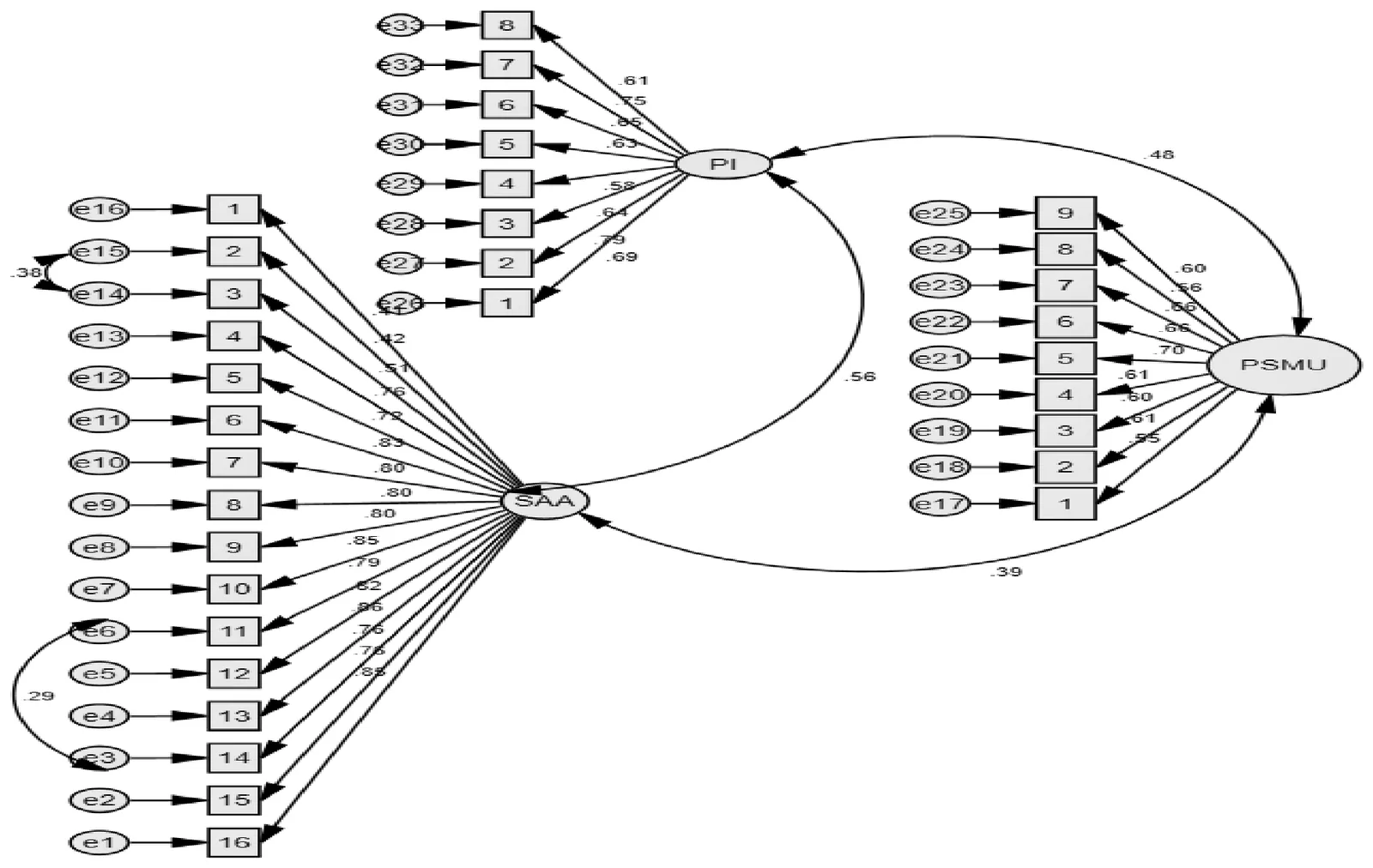
Three-Factor Confirmatory Factor Analysis (CFA) Measurement Model Showing Standardized Factor Loadings and Latent Factor Correlations. Note. Standardized estimates are presented. Correlations among latent factors were positive: SAA–PSMU = 0.39, PI–PSMU = 0.48, and SAA–PI = 0.56.

Before testing the structural model, a confirmatory factor analysis (CFA) was conducted to evaluate the three-factor measurement model consisting of SAA, PI, and PSMU. The findings are shown in Figure 2 and Table 2.

As shown in Table 2, the measurement model demonstrated an acceptable fit to the data, χ^2^ (490) = 1184.94, *p* < 0.001, χ^2^/df = 2.42, RMSEA = 0.05 (90% CI [0.05, 0.06]), CFI = 0.92, GFI = 0.87, AGFI = 0.85, IFI = 0.92, and TLI = 0.92. Overall, these findings indicated that the three-factor measurement model provided an acceptable fit to the observed data. The standardized factor loadings for the three-factor measurement model ranged from 0.41 to 0.86 for SAA, 0.55 to 0.70 for PSMU, and 0.58 to 0.79 for PI. These values indicate that all items had acceptable factor loadings on their respective latent constructs.

### 3.2. Mediation Model

**Figure 3:**
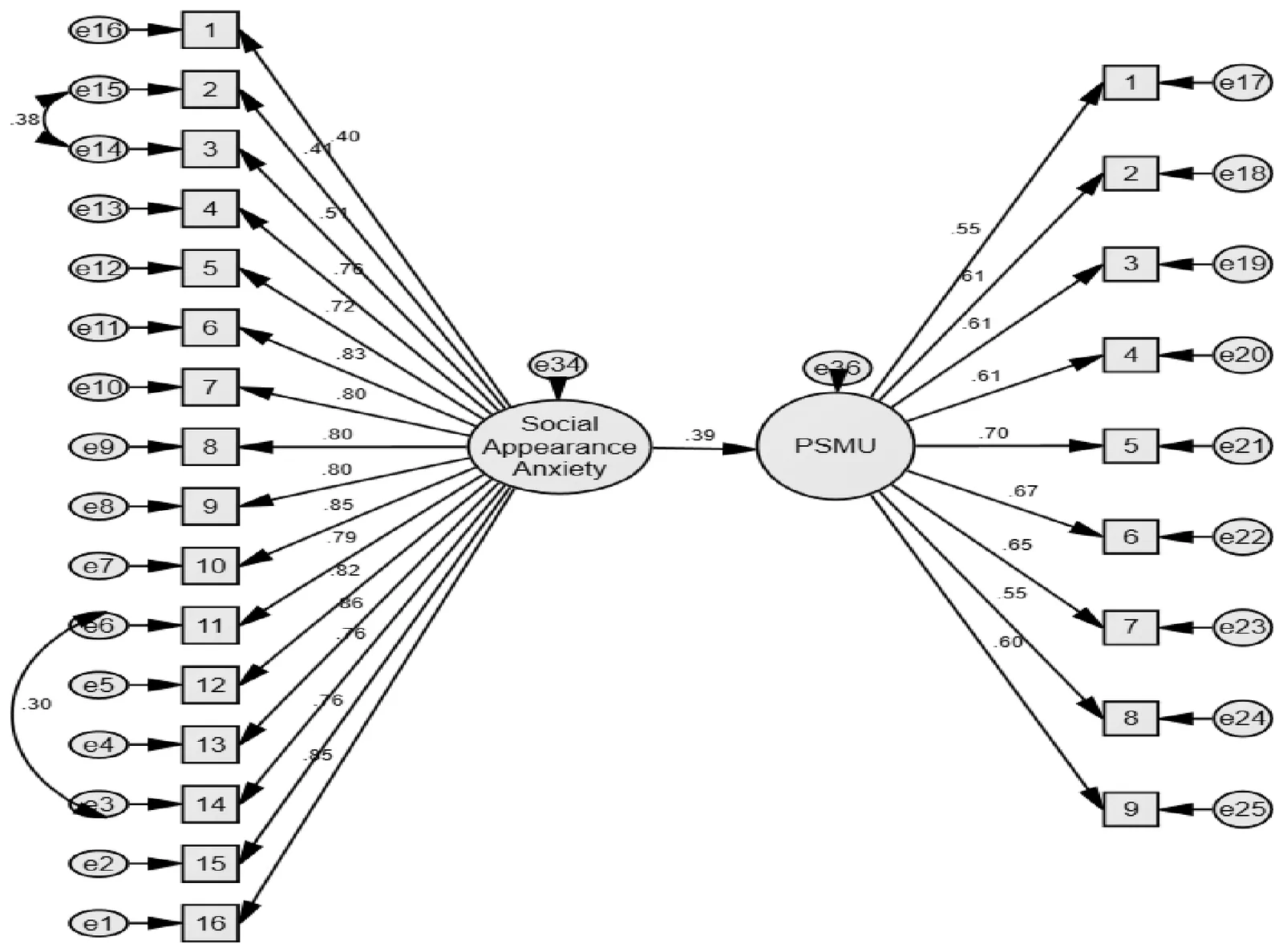
The Predictive Power of SAA on PSMU. Note. PSMU = Problematic Social Media Use.

The mediating role of PI was tested using AMOS 24. Skewness (0.389 to 0.599) and kurtosis (−0.129 to −0.095) coefficients were within ±1, satisfying the assumption of normality [50]. Z-scores indicated no univariate outliers. Prior to the structural equation modeling analyses, the data were screened for multivariate outliers and the assumption of multivariate normality. Multivariate outliers were identified using the Mahalanobis distance (D^2^) statistic. The Mahalanobis distance output, including the p1 and p2 probability values generated by AMOS, indicated several observations with significant multivariate distances. These observations were carefully examined and were considered to represent valid responses rather than data entry errors; therefore, they were retained in the analyses. Multivariate normality was assessed using Mardia’s multivariate kurtosis coefficient. The results yielded a multivariate kurtosis value of 308.83 with a critical ratio (c.r.) of 71.19, indicating a substantial violation of the assumption of multivariate normality. Given the violation of multivariate normality, the Bollen–Stine bootstrap procedure was employed to obtain more robust parameter estimates. The Bollen–Stine bootstrap test was statistically significant (*p* < 0.001), suggesting that the model did not perfectly reproduce the observed covariance matrix. However, because the chi-square statistic and the Bollen–Stine test are highly sensitive to large sample sizes, model adequacy was evaluated primarily using multiple goodness-of-fit indices rather than relying solely on the bootstrap significance test. The predictive power of SAA on PSMU was examined (Figure 3).

Figure 3 illustrates a positive and significant relationship between SAA and PSMU (*β* = 0.39, *p* < 0.001). Thus, SAA was significantly associated with PSMU, further supporting H1. Model fit indices are shown in Table 3.

**Figure 4:**
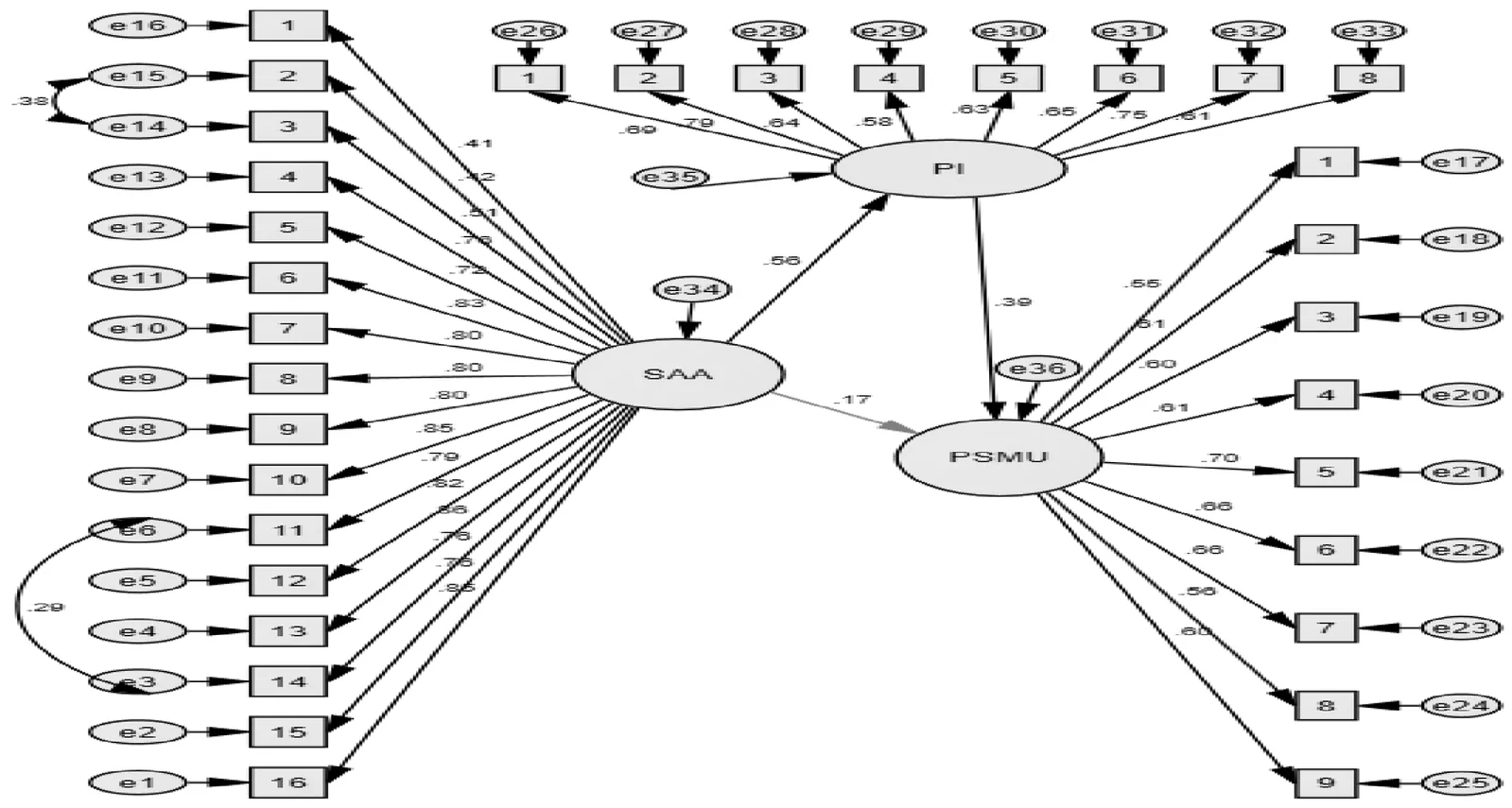
The Mediation Model.

As shown in Table 3, the fit indices were within the acceptable range. The chi-square test indicated that the model had an acceptable fit to the data (χ^2^ = 813.14, df = 272, *p* < 0.001). The χ^2^/df ratio (2.99) indicated an acceptable model fit, and the RMSEA = 0.06 (90% CI [0.059, 0.069]) suggested a good fit. The model fit indices, including CFI (0.93), IFI (0.93), and TLI (0.92), were within the acceptable range, whereas GFI (0.87) and AGFI (0.85) showed relatively lower values. Considering the overall pattern of fit indices [48,51], the tested direct-effect model demonstrated an acceptable fit to the data. Subsequently, PI was added to the model to examine its mediating role (H2). The results are presented in Figure 4.

Figure 4 illustrates that including PI significantly reduces the direct path coefficient between SAA and PSMU from 0.39 to 0.17. The significant indirect effect and the remaining significant direct effect suggest that PI partially mediates the relationship between SAA and PSMU. Fit indices for the mediation model were within acceptable ranges (Table 4).

The chi-square test was significant (χ^2^ = 1184.94, df = 490, *p* < 0.001). The χ^2^/df ratio (2.42) indicated an excellent fit, and the RMSEA (0.05, 90% CI [0.05, 0.06]) indicated an excellent fit. Since CFI (0.92), GFI (0.87), AGFI (0.85), TLI (0.92) and IFI (0.92) met the acceptable fit criteria [51,52], the tested mediation model demonstrated an acceptable fit to the observed data. The standardized path coefficients and bootstrap results are presented in Table 5.

Bootstrap analyses based on 5000 resamples indicated that the standardized total effect of SAA on PSMU was significant (*β* = 0.39, 95% CI [0.33–0.45]). After PI was included in the model, the standardized direct effect of SAA on PSMU remained significant (*β* = 0.17, 95% CI [0.09–0.25]). Furthermore, the standardized indirect effect of SAA on PSMU through PI was significant (*β* = 0.22, 95% CI [0.17–0.27]), indicating partial mediation.

The overall fit indices indicated that the proposed mediation model demonstrated an acceptable level of fit to the data. Specifically, the χ^2^/df ratio was 2.42, indicating excellent fit, while the RMSEA was 0.05 (90% CI [0.05, 0.06]), also reflecting excellent model fit. Furthermore, the incremental fit indices (CFI = 0.92, IFI = 0.92, and TLI = 0.92) and the absolute fit indices (GFI = 0.87 and AGFI = 0.85) were within acceptable ranges. Taken together, these findings suggest that, despite the violation of multivariate normality, the proposed structural model provided an acceptable representation of the observed data and was therefore considered appropriate for hypothesis testing.

### 3.3. Alternative Model Analysis

**Figure 5:**
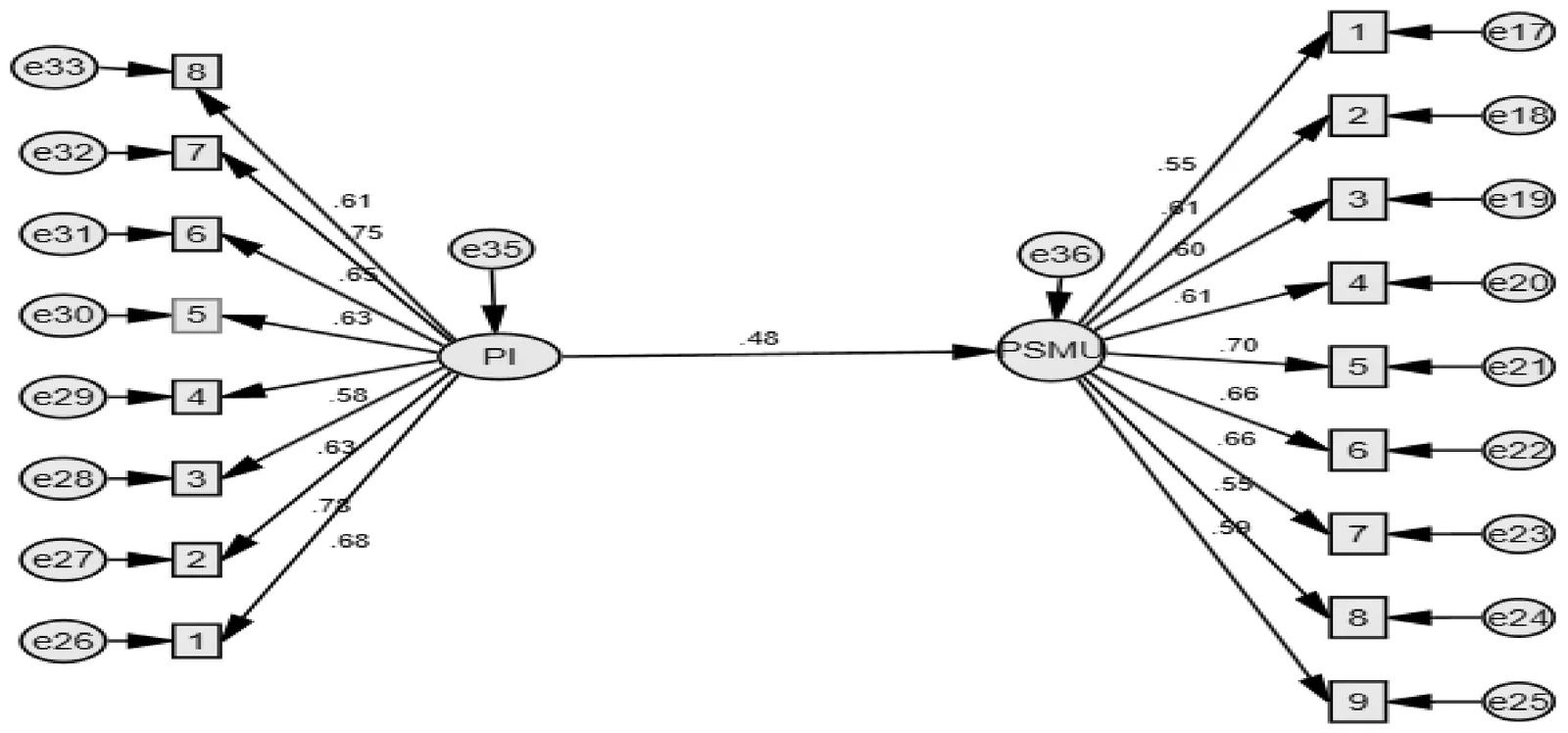
The Predictive Power of PI on PSMU.

Before the mediation analysis, the predictive level of PI on the PSMU was examined, and the findings were demonstrated in Figure 5.

Figure 5 illustrates a positive and significant relationship between PI and PSMU (*β* = 0.48, *p* < 0.001).

**Figure 6:**
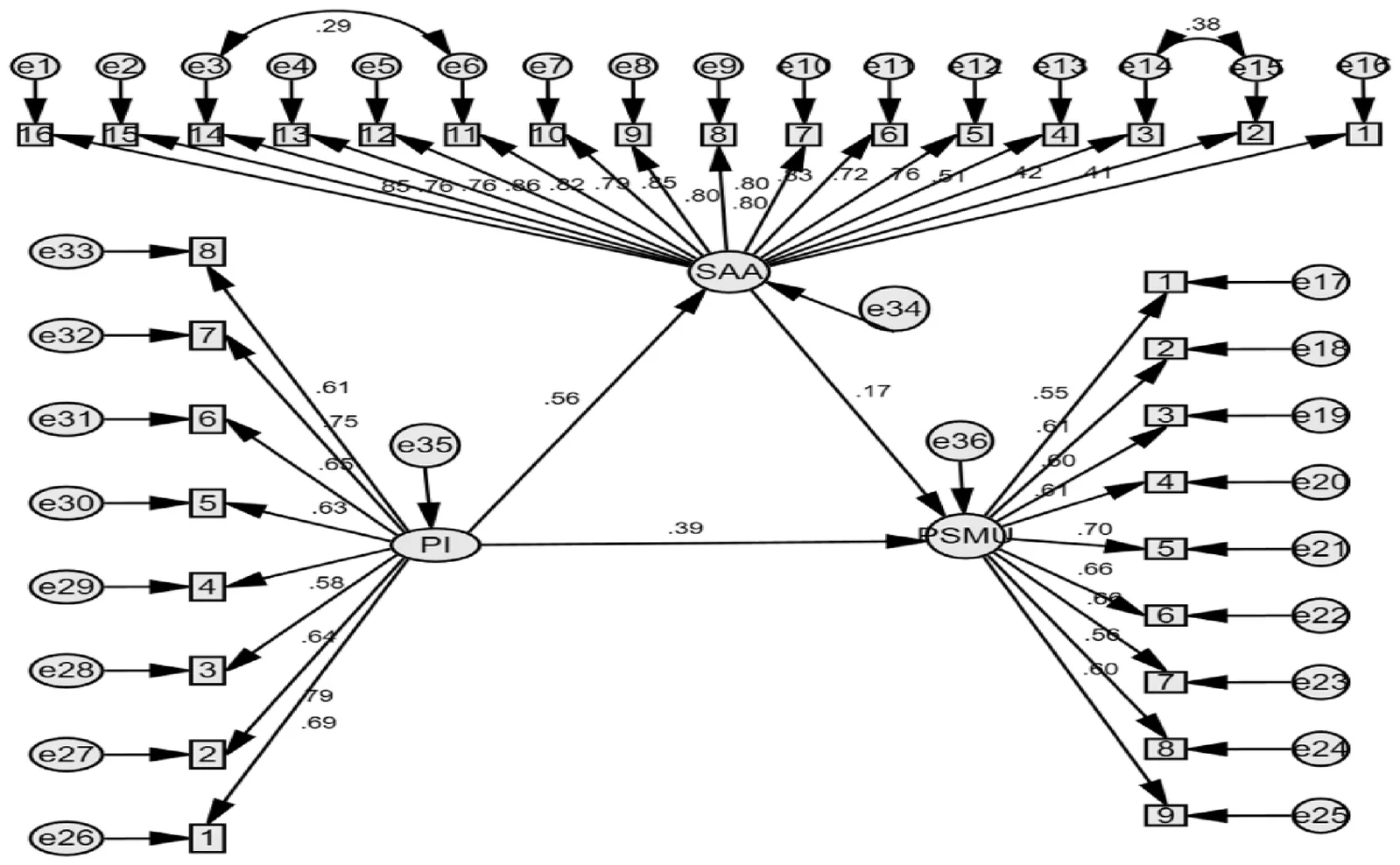
The Alternative Mediation Model.

Figure 6 illustrates the alternative structural model. The alternative model demonstrated an acceptable fit to the data, χ^2^(490) = 1184.942, *p* < 0.001, χ^2^/df = 2.418, RMSEA = 0.054, 90% CI [0.050, 0.059], CFI = 0.923, TLI = 0.917, GFI = 0.865 and AGFI = 0.845. In this model, PI positively and significantly predicted both SAA (*β* = 0.56, *p* < 0.001) and PSMU (*β* = 0.39, *p* < 0.001). In addition, SAA positively and significantly predicted PSMU (*β* = 0.17, *p* < 0.001).

Compared with the alternative model, the hypothesized model showed a greater reduction in the direct effect after the mediator was introduced. Specifically, the direct effect of SAA on PSMU decreased from *β* = 0.39 to *β* = 0.17. In contrast, the direct effect of PI on PSMU in the alternative model decreased from *β* = 0.48 to *β* = 0.39. This pattern suggests that PI explains a larger proportion of the association between SAA and PSMU than SAA explains the association between PI and PSMU.

## 4. Discussion

The main findings of this study, which aimed to examine the mediating role of psychological inflexibility in the relationship between PSMU and SAA among adolescents, are as follows: SAA among adolescents was found to be positively associated with PSMU and psychological inflexibility; psychological inflexibility partially mediated the relationship between SAA and PSMU; and PI emerged as significantly associated with PSMU among high school students. Furthermore, SAA showed significant direct and indirect associations with PSMU. However, given the study’s cross-sectional design, the proposed mediation model should be interpreted as explaining the relationships among the variables rather than establishing causal relationships.

Previous studies have found SAA to be positively associated with PSMU [53,54]. In addition, previous research has indicated that psychological flexibility is associated with appearance-related anxiety, with lower levels of psychological flexibility being related to greater appearance concerns [33]. Moreover, PI has been found to be associated with problematic digital behaviors, including PSMU [32]. Although previous studies have demonstrated associations between PI and PSMU, the present study contributes to this literature by examining whether PI may account for part of the association between SAA and PSMU within the framework of ACT, rather than focusing solely on the direct association between SAA and PSMU.

Adolescents with higher levels of SAA may experience greater PI, which may, in turn, be associated with higher levels of PSMU. This finding extends previous research by suggesting that PI may account for part of the association between appearance-related concerns and PSMU. Although previous studies have demonstrated associations between PI and problematic behaviors, the present study examined the potential role of PI in the association between SAA and PSMU, thereby contributing to a more comprehensive understanding of the factors associated with PSMU.

This study has certain limitations; one of these is that the sample does not fully represent the general population, which may limit the generalizability of the findings. Additionally, data were collected from participants using self-report methods; therefore, common method variance may have influenced the observed associations. Female students made up the vast majority of the study’s participants (68%). Another limitation is that gender was not included as a covariate in the model. Future studies should examine whether the findings remain consistent after controlling for gender. Although detailed information about each student’s age has not been collected, it is known that the study group includes students from every grade level in high school and that the students’ ages range from 14 to 18. The data were collected at a single point in time, and changes that may have occurred over time could not be examined. Another limitation is that the study did not include or control for offline factors, such as peer pressure or traditional media exposure. Therefore, unmeasured factors may have influenced the observed relationships among SAA, PI, and PSMU, and it remains unclear whether these relationships are specific to problematic social media use or reflect broader psychosocial processes. Future research should examine the proposed mediation model while controlling for these offline factors. Future research using longitudinal designs and involving different ethnic or age groups may provide further evidence regarding the relationships among these variables and improve the generalizability of the findings. Despite these limitations, the findings suggest that PI may be an important focus for future prevention and intervention research. Future longitudinal and experimental studies are needed to clarify this relationship.

## 5. Conclusions

This study examined the relationships among PSMU, PI, and SAA in an adolescent sample and provides new findings regarding the role of PI in the relationship between SAA and PSMU. Our findings showed that SAA was positively associated with PSMU and that PI significantly mediated this relationship. These findings contribute to the existing literature by highlighting the importance of psychological inflexibility in understanding PSMU among adolescents. Considering psychological inflexibility may provide a more comprehensive perspective for understanding PSMU among adolescents with higher levels of SAA. Therefore, future research with adolescents may benefit from further examining psychological inflexibility as a psychological process linking SAA and PSMU.

## Figures and Tables

**Table 1:** The Results of Correlation Analysis.

Variables	1	2	3	Mean	SD	Skewness	Kurtosis
PSMU	1			21.36	7.03	0.389	−0.129
PI	0.43 **	1		11.52	7.84	0.599	−0.198
SAA	0.35 **	0.51 **	1	39.24	15.12	0.594	−0.095

** *p* < 0.01. Note. SAA = Social Appearance Anxiety; PI = Psychological Inflexibility; PSMU = Problematic Social Media Use.

**Table 2:** Fit Indices for the Three-Factor Measurement Model.

Indices	Excellent Fit Criteria	Acceptable Fit Criteria	Model Fit Indices	Result
χ^2^/df	≤2.5	≤5	2.42	Acceptable fit
RMSEA	≤0.05	≤0.08	0.05	Acceptable fit
CFI	≥0.95	≥0.90	0.92	Acceptable fit
GFI	≥0.90	≥0.85	0.87	Acceptable fit
AGFI	≥0.90	≥0.85	0.85	Acceptable fit
IFI	≥0.95	≥0.90	0.92	Acceptable fit
TLI	≥0.95	≥0.90	0.92	Acceptable fit

**Table 3:** Model Fit Indices for the Initial Direct-Effect Model.

Indices	Excellent Fit Criteria	Acceptable Fit Criteria	Model Fit Indices	Result
χ^2^/df	≤2.5	≤5.00	2.99	Acceptable fit
RMSEA	≤0.05	≤0.08	0.06 (90% CI [0.059, 0.069])	Acceptable fit
CFI	≥0.95	≥0.90	0.93	Acceptable fit
GFI	≥0.90	≥0.85	0.87	Acceptable fit
AGFI	≥0.90	≥0.85	0.85	Acceptable fit
IFI	≥0.95	≥0.90	0.93	Acceptable fit
TLI	≥0.95	≥0.90	0.92	Acceptable fit

**Table 4:** Model Fit Indices for the Mediation Model.

Fit Indices	Excellent Fit Cutoff	Acceptable Fit Cutoff	Model Fit Indices	Result
χ^2^/df	≤2.50	≤5.00	2.42	Excellent fit
RMSEA	≤0.05	≤0.08	0.05 (90% CI [0.05, 0.06])	Excellent fit
CFI	≥0.95	≥0.90	0.92	Acceptable fit
GFI	≥0.90	≥0.85	0.87	Acceptable fit
AGFI	≥0.90	≥0.85	0.85	Acceptable fit
IFI	≥0.95	≥0.90	0.92	Acceptable fit
TLI	≥0.95	≥0.90	0.92	Acceptable fit

**Table 5:** The Results of Bootstrap Analyses.

Path	Unstandardized (*β*)	SE	Standardized (*β*)	SE	Unstandardized 95% CI		Standardized 95% CI		*p*
					Lower	Upper	Lower	Upper	
Total Effect SAA → PSMU	0.22	0.03	0.39	0.05	0.18	0.26	0.33	0.45	(*p* = 0.001)
Direct Effect SAA → PSMU	0.10	0.04	0.17	0.07	0.05	0.15	0.09	0.25	(*p* = 0.001)
Indirect Effect SAA → PI → PSMU	0.12	0.02	0.22	0.04	0.09	0.15	0.17	0.27	(*p* = 0.001)

## Data Availability

The data that support the findings of this study are available from the corresponding author upon reasonable request.

## References

[1] Statista (2025). Internet and Social Media Users in the World 2025 [Statista Statistic]. https://www.statista.com/statistics/617136/digital-population-worldwide.

[2] Guan H., Peng S., Liu Z., Sun H., Wu H., Yao X., Chen Z., Yang X. (2025). Effect of emotional intelligence on problematic social media use among Chinese college students: Mediating role of social exclusion and experiential avoidance. Front. Psychiatry.

[3] Boursier V., Gioia F., Griffiths M.D. (2020). Do selfie-expectancies and social appearance anxiety predict adolescents’ problematic social media use?. Comput. Hum. Behav..

[4] Nagata J.M., Lee C.M., Hur J.O., Baker F.C. (2025). What we know about screen time and social media in early adolescence: A review of findings from the Adolescent Brain Cognitive Development Study. Curr. Opin. Pediatr..

[5] Larsen B., Luna B. (2018). Adolescence as a neurobiological critical period for the development of higher-order cognition. Neurosci. Biobehav. Rev..

[6] Jiang Y., Bai X., A L., Liu Y., Li M., Liu G. (2016). Problematic social networks usage of adolescent. Adv. Psychol. Sci..

[7] Wegmann E., Stodt B., Brand M. (2015). Addictive use of social networking sites can be explained by the interaction of Internet use expectancies, Internet literacy, and psychopathological symptoms. J. Behav. Addctn..

[8] Wan C. (2009). Gratifications & Loneliness as Predictors of Campus-SNS Websites Addiction & Usage Pattern Among Chinese College Students. Doctoral Dissertation.

[9] Faverio M., Sidoti O. (2024). Teens, Social Media and Technology 2024.

[10] Lenhart A., Duggan M., Perrin A., Stepler R., Rainie H., Parker K. (2015). Teens, Social Media and Technology Overview 2015: Smartphones Facilitate Shifts in Communication Landscape for Teens.

[11] Boer M., van den Eijnden R.J.J.M., Boniel-Nissim M., Wong S.L., Inchley J.C., Badura P., Craig W.M., Gobina I., Kleszczewska D., Klanšček H.J. (2020). Adolescents’ intense and problematic social media use and their well-being in 29 countries. J. Adolesc. Health.

[12] Marino C., Gini G., Vieno A., Spada M.M. (2018). The associations between problematic Facebook use, psychological distress and well-being among adolescents and young adults: A systematic review and meta-analysis. J. Affect. Disord..

[13] Williams M., Lewin K.M., Meshi D. (2024). Problematic use of five different social networking sites is associated with depressive symptoms and loneliness. Curr. Psychol..

[14] Lopes L.S., Valentini J.P., Monteiro T.H., Costacurta M.C.d.F., Soares L.O.N., Telfar-Barnard L., Nunes P.V. (2022). Problematic social media use and its relationship with depression or anxiety: A systematic review. Cyberpsychology Behav. Soc. Netw..

[15] Wang J., Wang N., Liu Y., Zhou Z. (2025). Experiential avoidance, depression, and difficulty identifying emotions in social network site addiction among Chinese university students: A moderated mediation model. Behav. Inf. Technol..

[16] Hart T.A., Flora D.B., Palyo S.A., Fresco D.M., Holle C., Heimberg R.G. (2008). Development and examination of the Social Appearance Anxiety Scale. Assessment.

[17] Alden L.E., Taylor C.T. (2004). Interpersonal Processes in Social Phobia. Clin. Psychol. Rev..

[18] Coles M.E., Phillips K.A., Menard W., Pagano M.E., Fay C., Weisberg R.B., Stout R.L. (2006). Body dysmorphic disorder and social phobia: Cross-sectional and prospective data. Depress. Anxiety.

[19] Ruan J., Yu R., Zhao Y., Xie L., Mei Y. (2025). Body talk on social networking sites and appearance anxiety among college students: The mediating role of self-objectification and moderating role of gender. Front. Psychol..

[20] Zhang Z., Zhou M. (2024). The impact of social media information exposure on appearance anxiety in young acne patients: A moderated chain mediation model. Front. Psychol..

[21] Söyünmez S., Efe Y.S., Kudubeş A.A., Caner N. (2024). The predictors of objectified body consciousness among adolescents: Social appearance anxiety and social media use. Arch. Psychiatr. Nurs..

[22] Caner N., Efe Y.S., Başdaş Ö. (2022). The contribution of social media addiction to adolescent life: Social appearance anxiety. Curr. Psychol..

[23] Ayar D., Özalp Gerçeker G., Özdemir E.Z., Bektaş M. (2018). The effect of problematic Internet use, social appearance anxiety, and social media use on nursing students’ nomophobia levels. CIN Comput. Inform. Nurs..

[24] Tingrong Y., Gen Z. (2025). Magic mirror, who is the fairest one of all? Testing the mediating effect between short-video social media exposure and appearance anxiety. BMC Psychol..

[25] Wu Y., Xue Y., Zhao X., Han S., Wu W. (2024). Unravelling the veil of appearance anxiety: Exploring social media use among Chinese young people. BMC Psychol..

[26] Ekinci N., Akat M. (2023). The relationship between anxious-ambivalent attachment and social appearance anxiety in adolescents: The serial mediation of positive youth development and Instagram addiction. Psychol. Rep..

[27] Sarda E., El-Jor C., Shankland R., Hallez Q., Patiram D., Nguyen C., Duflos N., Durand Y., Del Pozo G., Ezan P. (2025). Social media use and roles of self-objectification, self-compassion, and body image concerns: A systematic review. J. Eat. Disord..

[28] Fardouly J., Diedrichs P.C., Vartanian L.R., Halliwell E. (2015). Social comparisons on social media: The impact of Facebook on young women’s body image concerns and mood. Body Image.

[29] Hayes S.C., Luoma J.B., Bond F.W., Masuda A., Lillis J. (2006). Acceptance and commitment therapy: Model, processes and outcomes. Behav. Res. Ther..

[30] Hayes S.C., Strosahl K.D., Wilson K.G. (2012). Acceptance and Commitment Therapy: The Process and Practice of Mindful Change.

[31] Chou W.P., Lee K.H., Ko C.H., Liu T.L., Hsiao R.C., Lin H.F., Yen C.F. (2017). Relationship between psychological inflexibility and experiential avoidance and Internet addiction: Mediating effects of mental health problems. Psychiatry Res..

[32] Liu C., Rotaru K., Chamberlain S.R., Ren L., Fontenelle L.F., Lee R.S.C., Suo C., Raj K., Yücel M., Albertella L. (2022). The moderating role of psychological flexibility on the association between distress-driven impulsivity and problematic Internet use. Int. J. Environ. Res. Public Health.

[33] Shepherd L., Reynolds D.P., Turner A., O’Boyle C.P., Thompson A.R. (2019). The role of psychological flexibility in appearance anxiety in people who have experienced a visible burn injury. Burns.

[34] Karaaziz M., Razzaghi P., Keskindag B., Güney H. (2024). Brief report: A feasibility study of acceptance and commitment therapy in group format for social appearance anxiety. Curr. Psychol..

[35] Morton C., Mooney T.A., Lozano L.L., Adams E.A., Makriyianis H.M., Liss M. (2020). Psychological inflexibility moderates the relationship between thin-ideal internalization and disordered eating. Eat. Behav..

[36] Zucchelli F., White P., Williamson H., VTCT Foundation Research Team at the Centre for Appearance Research (2020). Experiential avoidance and cognitive fusion mediate the relationship between body evaluation and unhelpful body image coping strategies in individuals with visible differences. Body Image.

[37] Tras Z., Öztemel K., Baltacı U.B. (2019). Role of problematic Internet use, sense of belonging and social appearance anxiety in Facebook use intensity of university students. Int. Educ. Stud..

[38] Kenny U., O’Malley-Keighran M.-P., Molcho M., Kelly C. (2017). Peer influences on adolescent body image: Friends or foes?. J. Adolesc. Res..

[39] Yiğit H. (2022). Ergenlerin Sosyal Medya Bağımlılık Düzeyleri ile Sosyal Görünüş Kaygıları ve Yeme Tutumlari Arasindaki Ilişkinin Incelenmesi Yüksek Lisans Tezi.

[40] Twohig M.P., Levin M.E. (2017). Acceptance and Commitment Therapy for Anxiety Disorders: A Practitioner’s Treatment Guide to Using Mindfulness, Acceptance, and Values-Based Behavior Change.

[41] Yang H., Quek F.Y.X., Yap S.X.H., Tng G.Y.Q., Ng G.R. (2026). A stress-induced digital escapism framework for understanding the link between stress and problematic social media use. Behav. Sci..

[42] Doğan T. (2010). Sosyal görünüş kaygısı ölçeği’nin (SGKÖ) Türkçe uyarlaması: Geçerlik ve güvenirlik çalışması. Hacet. Üniversitesi Eğitim Fakültesi Derg..

[43] Greco L.A., Lambert W., Baer R.A. (2008). Psychological inflexibility in childhood and adolescence: Development and evaluation of the Avoidance and Fusion Questionnaire for Youth. Psychol. Assess..

[44] Büyüköksüz E., Erözkan A. (2019). Kaçınma ve birleşme ölçeği–gençler 8 (KBÖ-G8): Türk kültüründe faktör yapısı ve güvenirlik çalışmaları. J. Cogn. Behav. Psychother. Res..

[45] Özgenel M., Canpolat Ö., Ekşi H. (2019). Ergenler için sosyal medya bağımlılığı ölçeği (ESMBÖ): Geçerlik ve güvenirlik çalışması. Addicta Turk. J. Addctn..

[46] Öz Tatacak S., Yar Z., Elibol Z., Adıgüzel D., Koşar Z.N. (2026). Adolescents’ problematic social media use and socioemotional well-being: A structural equation modeling approach. Child Indic. Res..

[47] Byrne B.M. (2016). Structural Equation Modeling with AMOS: Basic Concepts, Applications, and Programming.

[48] Kline R.B. (2023). Principles and Practice of Structural Equation Modeling.

[49] Schoemann A.M., Boulton A.J., Short S.D. (2017). Determining power and sample size for simple and complex mediation models. Soc. Psychol. Pers. Sci..

[50] Çokluk Ö., Şekercioğlu G., Büyüköztürk Ş. (2012). Sosyal Bilimler Için Çok Değişkenli Istatistik: SPSS Ve LISREL Uygulamaları.

[51] Hu L.T., Bentler P.M. (1999). Cutoff criteria for fit indexes in covariance structure analysis: Conventional criteria versus new alternatives. Struct. Equ. Model. Multidiscip. J..

[52] Kline R.B. (2016). Principles and Practice of Structural Equation Modeling.

[53] Aslan H.R., Tolan Ö.Ç. (2022). Social appearance anxiety, automatic thoughts, psychological well-being and social media addiction in university students. Int. Educ. Stud..

[54] Özok H.İ., Kaya A., Dinçer R., Yıldırım M. (2025). Associations between social appearance anxiety, problematic social media use, selfitis behavior, and adaptable self: A moderated-mediation model. Psihologija.

